# Metabolic syndrome and risk of incident chronic pancreatitis: a prospective cohort study

**DOI:** 10.3389/fnut.2026.1817098

**Published:** 2026-04-29

**Authors:** Ruoyi Zhang, Zewen Zhang, Zhen Ding, Zhenxiong Liu

**Affiliations:** 1 Department of Gastroenterology, Tangdu Hospital, Fourth Military Medical University, Xi'an, China; 2 Department of Gastroenterology, The First Affiliated Hospital, Sun Yat-Sen University, Guangzhou, China

**Keywords:** chronic pancreatitis, high blood pressure, high-density lipoprotein cholesterol, metabolic syndrome, obesity, type 2 diabetes mellitus, UK biobank

## Abstract

**Background and aim:**

The association between metabolic syndrome (MetS) and chronic pancreatitis (CP) remains unclear. This study aimed to examine whether MetS is independently associated with an increased risk of CP.

**Methods:**

We analyzed data from 349,995 participants in the UK Biobank who were free of CP at baseline. MetS was defined according to NCEP-ATP III criteria. Incident CP cases were identified via hospital admissions and death registries using ICD codes. Cox proportional hazards models were used to estimate hazard ratios (HRs) with sequential adjustment for demographic, lifestyle, clinical, and inflammatory factors. Subgroup, nonlinear, mediation, and sensitivity analyses were performed to assess robustness.

**Results:**

Over a median follow-up of 18 years, 623 incident CP cases occurred. MetS was significantly associated with increased CP risk after full adjustment (HR 2.02, 95% CI 1.64–2.49). A clear dose–response relationship was observed between the number of MetS components and CP risk. Among individual components, hyperglycemia (HR 2.59) and elevated waist-to-hip ratio (Q4 vs. Q1 HR 2.78) were the strongest predictors. Subgroup analyses showed stronger associations in younger individuals, females, never smokers, and current drinkers. Systemic inflammation, particularly via neutrophils and CRP, mediated approximately 8 and 3% of the association, respectively. Sensitivity analyses confirmed the robustness of these findings.

**Conclusion:**

MetS is an independent risk factor for CP, with risk rising as the number of MetS components increases. Hyperglycemia and central adiposity are key drivers. Managing metabolic health may help reduce CP risk.

## Introduction

Chronic pancreatitis (CP) is a progressive fibro-inflammatory pancreatic disorder with a significant global health burden. Epidemiologically, CP shows distinct geographic patterns: in Western populations, annual incidence ranges from 3 to 10 per 100,000 person-years, with alcohol accounting for 60%–70% of cases. In contrast, many Asian regions report higher proportions of idiopathic and tropical pancreatitis, reflecting differing genetic and environmental influences (1).

The etiology of CP is multifactorial and often involves a complex interplay of environmental, metabolic, and genetic factors. Long-term heavy alcohol use is a well-established major risk factor, with risk increasing in a dose-dependent manner (2). Tobacco smoking independently doubles or triples the risk and exhibits a synergistic effect with alcohol (3). Hereditary factors play a significant role, with pathogenic variants in genes such as *PRSS1*, *SPINK1*, and *CFTR* contributing to familial and early-onset forms (4). Emerging evidence highlights the importance of metabolic disturbances, including obesity, and dyslipidemia, as increasingly recognized contributors to CP pathogenesis (5, 6). Furthermore, recurrent acute pancreatitis is a critical clinical pathway, with approximately 30% of cases progressing to chronic disease (7).

Metabolic syndrome (MetS) is a cluster of interrelated cardiometabolic conditions, including central obesity, hypertension, dysglycemia (insulin resistance or elevated fasting glucose), and dyslipidemia (elevated triglycerides and/or low high-density lipoprotein cholesterol) (8). It represents a state of systemic inflammation, oxidative stress, and hormonal dysregulation. While MetS is a well-recognized risk factor for cardiovascular disease (9), type 2 diabetes (10), and certain cancers (11), its relationship with pancreatic diseases has gained increasing attention. Notably, MetS and its components—particularly hypertriglyceridemia, insulin resistance, and visceral adiposity—have been associated with acute pancreatitis, both in terms of increased incidence and severity (12). Furthermore, epidemiological and clinical studies suggest that metabolic dysregulation may contribute to pancreatic ductal adenocarcinoma risk, possibly through chronic inflammation, hyperinsulinemia, and altered adipokine profiles (13).

Despite these established links with other pancreatic conditions, the association between MetS and chronic pancreatitis remains underexplored in systematic research. Mechanistically, MetS may influence CP pathogenesis through several pathways: chronic low-grade inflammation may promote pancreatic fibrogenesis (14); hypertriglyceridemia can induce pancreatic acinar injury; and insulin resistance may alter pancreatic repair mechanisms (15). Additionally, visceral adipose tissue releases pro-inflammatory cytokines and free fatty acids, which could accelerate pancreatic injury and stellate cell activation (16).

This study applies an integrated statistical approach to assess the relationship between metabolic syndrome and chronic pancreatitis. Cox regression models will estimate adjusted hazard ratios, with subgroup analyses evaluating effect modification. Nonlinear dose–response patterns will be examined using restricted cubic splines. Mediation analysis will quantify the contribution of inflammatory biomarkers. Sensitivity analyses, including multiple imputation and exclusion of early cases, will test result robustness. This integrated analytical framework aims to comprehensively assess the epidemiological link, potential biological pathways, and consistency of the association between metabolic syndrome and chronic pancreatitis risk.

## Methods

### Study population

**Figure 1:**
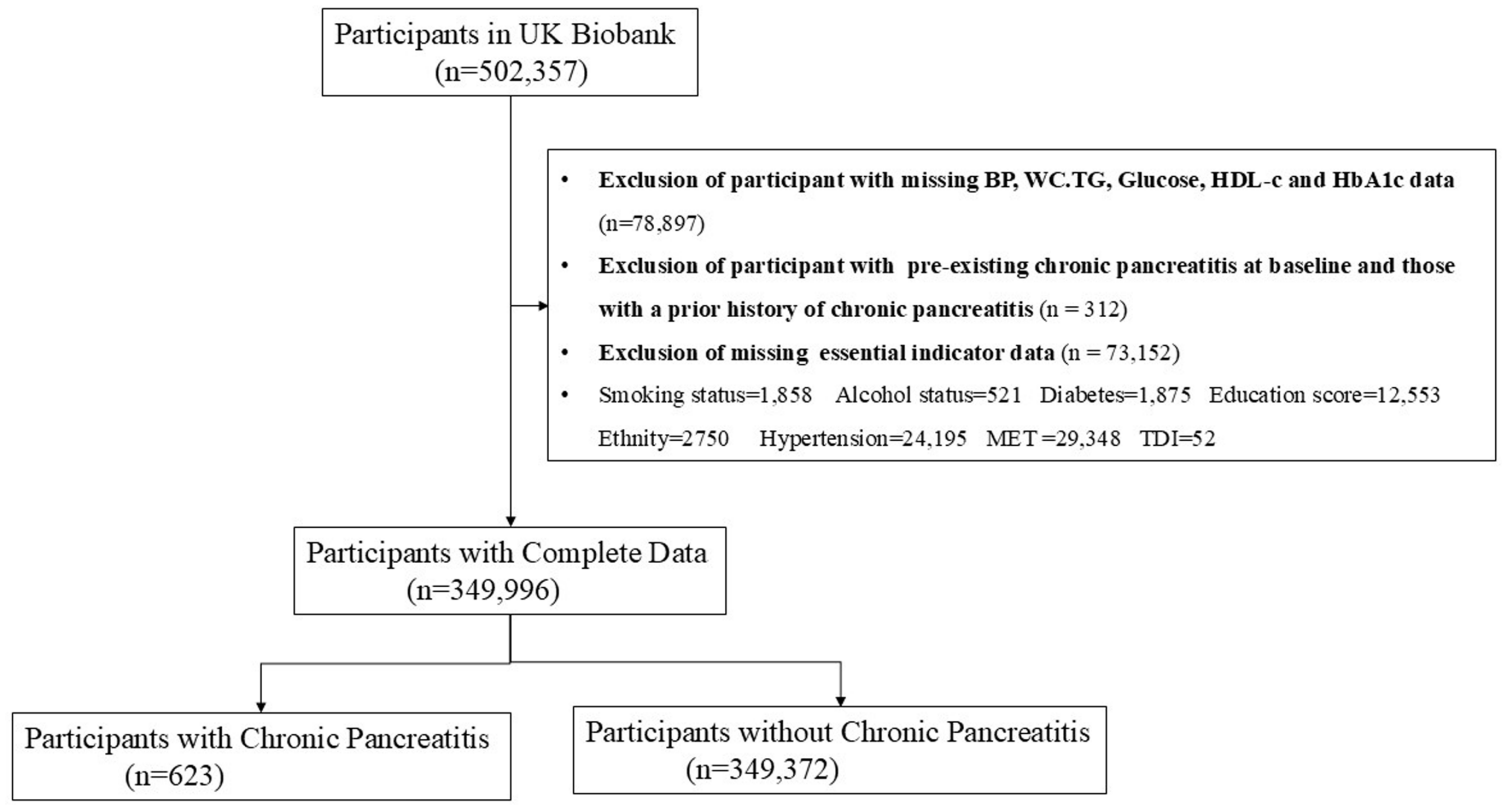
Flowchart of participant selection.

This analysis utilized the UK Biobank, a large-scale prospective cohort. Between 2006 and 2010, it recruited over half a million participants aged 40–69 years. The resource contains detailed longitudinal data, including questionnaires, physical measurements, biological samples, genomic profiles, and health records. Ethical approval was granted by the Northwest Multicenter Research Ethics Committee (11/NW/0382) (17). All participants provided informed written consent for data usage and linkage, following the Declaration of Helsinki (18). Access was obtained under application 533,022. From the initial cohort, we sequentially applied the following exclusion criteria: (1) Individuals with a history of chronic pancreatitis at baseline (*n* = 312). (2) Participants with missing data on key cardiometabolic indicators (blood pressure, waist circumference, triglycerides, glucose, high-density lipoprotein cholesterol, or glycated hemoglobin) (*n* = 78,897). (3) Participants missing other essential covariate data (*n* = 73,152). After applying these exclusions, the final analytical sample comprised 349,995 subjects. Figure 1 outlines the complete participant selection process.

### Assessment of metabolic syndrome traits

Metabolic syndrome was diagnosed according to the National Cholesterol Education Program Adult Treatment Panel III (NCEP-ATP III) criteria, requiring the presence of at least three of the following five components (19): (1) central obesity, defined as a waist circumference (WC) > 102 cm in men or >88 cm in women (Field ID [48]); (2) hypertension, defined as systolic blood pressure (SBP) ≥ 130 mmHg (Field ID [4080]), diastolic blood pressure (DBP) ≥ 85 mmHg (Field ID [4079]), or current use of antihypertensive medication (Field ID [6177]); (3) dyslipidemia, specifically low high-density lipoprotein cholesterol (HDL-C), defined as <1.0 mmol/L in men or <1.3 mmol/L in women (Field ID [30760]), or current use of lipid-lowering medication (Field ID [6177]); (4) hyperglycemia, defined as glycated hemoglobin (HbA1c) ≥ 42 mmol/mol (Field ID [30750]), current use of glucose-lowering medication (Field ID [6177]), or a previous diagnosis of type 2 diabetes (Field IDs [2443] and [2976]); and (5) hypertriglyceridemia, defined as triglyceride (TG) level ≥1.7 mmol/L (Field ID [30870]). For a comprehensive assessment of adiposity, body fat percentage was measured using bioelectrical impedance analysis (BIA) with the Tanita BC418MA body composition analyzer (20) (Field ID [23099]), and waist-to-hip ratio (WHR) was calculated from measured WC (Field ID [48]) and hip circumference (Field ID [49]). Resting systolic and diastolic blood pressures were measured twice using an Omron HBP-1300 digital monitor after a 5-min seated rest, and the average of the two readings was used (21). All biochemical assays (HDL-C, HbA1c, TG) were performed on blood samples collected at the baseline assessment visit (22). Medication use was identified from verbal interview data and coded under Field ID [6177].

### Assessment of chronic pancreatitis

Participants with chronic pancreatitis were identified from linked hospital admission and death registry data within the UK Biobank. Case status was assigned based on the presence of any of the following International Classification of Diseases (ICD) codes: ICD-10 codes K86.0 or K86.1, or ICD-9 code 577.1. These codes were extracted from the relevant UK Biobank fields for diagnoses (Field 41,270, 41,271) and cause of death (Field 40,001, 40,002). The UK Biobank primary care data were not used because they record code entry dates rather than true disease onset, preventing accurate determination of CP timing relative to baseline. Additionally, these data are already integrated into the ICD-9/ICD-10 data used here, and separate analysis would cause duplication.

### Assessment of covariates

In our multivariable analyses, we adjusted for an extensive set of potential confounders using data collected during the UK Biobank baseline assessment. The covariates were selected to address confounding across demographic, socioeconomic, anthropometric, lifestyle, metabolic, inflammatory, and clinical domains, and included the following: (1) Sociodemographic factors: chronological age (Field 21,003), biological sex (Field 31), self-reported ethnic background (Field 21,000), area-level socioeconomic deprivation (Townsend deprivation index [TDI]; Field 189), and educational attainment (Field 6,138) as a proxy for individual socioeconomic position. (2) Anthropometric measures: body mass index (BMI; derived from standing height [Field 50] and weight [Field 21,002]). (3) Lifestyle and behavioral factors: smoking status (categorized as never, previous, or current; Field 20,116), alcohol consumption status (never, previous, or current; Field 20,117), habitual sleep duration per 24-h period (Field 1,160), physical activity level (expressed as total metabolic equivalent task [MET] minutes per week, derived from International Physical Activity Questionnaire parameters (23) in Fields 22,038 and 22,039), and estimated daily energy intake in kilocalories (Field 100,002) from the 24-h dietary assessment.(4) Laboratory biomarkers: measured from fasting blood samples, including systemic inflammatory markers—C-reactive protein (CRP; Field 30,710) and a full white blood cell differential (lymphocyte count [Field 30,220] and percentage [Field 30,200], monocyte count [Field 30,130] and percentage [Field 30,110], neutrophil count [Field 30,140] and percentage [Field 30,120], and total white blood cell count [Field 30,000])—as well as additional hematological and biochemical parameters: hemoglobin concentration (Hb; Field 30,020), hematocrit (Hct; Field 30,030), platelet count (Field 30,080), total bilirubin (Field 30,840), and serum calcium (Field 30,680). (5) Prior clinical events: ascertained via linked electronic health records, including history of acute pancreatitis (AP) (identified using ICD-10 codes K85, K85.0–K85.3, K85.8–K85.9 and ICD-9 code 577.0) and documented gallstone disease (GSD) (identified using ICD-10 codes K80.0–K80.5, K80.8 and ICD-9 codes 574.0–574.5, 574.8–574.9) from hospital inpatient and mortality registries. This comprehensive adjustment strategy was implemented to minimize residual confounding, thereby improving the internal validity of our estimates and strengthening causal inference regarding the observed associations.

### Statistical analysis

The baseline characteristics of the study population were summarized using appropriate descriptive statistics based on the distribution and nature of the variables. For continuous variables, we assessed normality using the Shapiro–Wilk test and visual inspection of Q-Q plots. Normally distributed variables were presented as mean ± standard deviation (SD), while non-normally distributed variables were summarized as median with interquartile range (IQR). Categorical variables were expressed as frequencies with percentages (*n*, %). Between-group comparisons were conducted using standardized statistical tests. For continuous variables, we employed Student’s *t*-test for normally distributed variables and the Mann–Whitney *U* test for non-normally distributed variables when comparing two groups. For comparisons across multiple groups, we utilized one-way analysis of variance (ANOVA) for normally distributed variables and the Kruskal-Wallis test for non-normally distributed variables. Post-hoc analyses were performed.

The association between the primary exposure and the incidence of the outcome was assessed using Cox proportional hazards regression models. A sequential covariate adjustment approach was employed across three nested models to progressively account for potential confounding factors. Model 1 estimated the crude association, including only the primary exposure variable. Model 2 extended Model 1 by adjusting for age, sex, area-level socioeconomic deprivation, smoking status, alcohol consumption status, educational attainment, a prior history of AP, a prior history of GSD, BMI, and estimated daily energy intake. Model 3 further built upon Model 2 by incorporating a range of laboratory biomarkers, including serum calcium, C-reactive protein, lymphocyte percentage, monocyte count, neutrophil count and percentage, total white blood cell count, Hct, and Hb. The proportional hazards assumption was tested using Schoenfeld residuals, and no significant violations were observed. Person-time of follow-up was calculated from the date of the baseline assessment until the date of the first occurrence of the outcome event, death, loss to follow-up, or the administrative end of the study period, whichever came first.

To examine potential non-linear relationships between components of metabolic syndrome—specifically WC, HDL-c, TG, HbA1c, SBP, and DBP—and the risk of chronic pancreatitis, we conducted restricted cubic spline (RCS) analyses based on fully adjusted Cox proportional hazards models (Model 3). All models included adjustment for sociodemographic factors, lifestyle behaviors, clinical comorbidities, and comprehensive laboratory biomarkers. RCS analyses were implemented in R using the rms package, with three knots positioned at the 10th, 50th, and 90th percentiles of each exposure variable’s distribution. The linearity assumption was formally assessed using likelihood ratio tests, comparing a model containing only a linear term to one incorporating spline terms. A statistically significant *p*-value for non-linearity (*p* < 0.05) was interpreted as evidence of a non-linear association. For exposures demonstrating significant deviation from linearity, the inflection point—defined as the value at which the hazard ratio (HR) curve exhibited a meaningful change in slope—was identified by locating the point of maximum curvature on the fitted spline function. Results are presented graphically as smoothed HR curves with 95% confidence intervals, using the median value of each respective exposure as the reference (HR = 1) (24).

To evaluate potential effect modification and assess the robustness of the primary associations, we performed subgroup analyses based on the fully adjusted model. Prespecified stratification factors included: age (<60 vs. ≥60 years), sex, smoking status (never, previous, current), alcohol consumption (never, previous, current), TDI (categorized by average), education score (categorized by average), history of AP (yes vs. no), history of GSD (yes vs. no), BMI categories (<18.5, 18.5–25, ≥25 kg/m^2^), and LDL cholesterol (<4 vs. ≥4 mmol/L). Continuous variables were categorized using clinically relevant or sample median cut-offs. Effect modification was formally tested by including an interaction term between metabolic syndrome and each subgroup variable in separate Cox models, with significance assessed via likelihood ratio tests (two-sided interaction *p*-value <0.05 considered statistically significant). Subgroup-specific HRs with 95% confidence intervals (CIs) are presented along with the corresponding *p*-values for interaction.

To explore whether systemic inflammation mediates the association between metabolic syndrome and chronic pancreatitis, we conducted a formal mediation analysis within the framework of the fully adjusted Cox model. The analysis was performed in four sequential steps using a counterfactual-based approach (25). First, we estimated the total effect of metabolic syndrome on chronic pancreatitis incidence in a fully adjusted Cox proportional hazards model. Second, we evaluated path a (the association between metabolic syndrome and each inflammatory mediator) using multivariable linear regression models adjusted for all covariates. Third, we assessed path b (the association between each inflammatory mediator and chronic pancreatitis risk) by fitting Cox models that included both metabolic syndrome and the inflammatory mediator, along with all covariates. The indirect (mediated) effect was estimated using the product-of-coefficients method (a × b). Statistical significance was determined using percentile bootstrap confidence intervals based on 1,000 resamples. The proportion mediated was calculated as the ratio of the indirect effect to the total effect: (a × b)/total effect. In the mediation analysis, the Cox proportional hazards models were adjusted for the following covariates: age, sex, TDI, smoking status, alcohol drinker status, education score, history of AP, history of GSD, BMI, energy intake, calcium, Hct, and Hb.

To evaluate the robustness of our primary findings, we conducted a series of sensitivity analyses employing alternative analytical approaches and population subsets across all three nested Cox models (Models 1–3). First, to address potential selection bias due to missing data, we performed multiple imputation using chained equations (MICE). Twenty complete datasets were generated by imputing missing covariate values based on the observed data structure. The Cox proportional hazards models for all three adjustment levels were re-estimated across these imputed datasets, and results were pooled according to Rubin’s rules (26). Second, to examine the potential influence of competing risks and informative censoring, we repeated the analyses for Models 1–3 after excluding participants who either died or were lost to follow-up during the study period. Third, to mitigate potential reverse causality whereby undiagnosed chronic pancreatitis at baseline could influence metabolic syndrome status, we sequentially excluded participants who developed chronic pancreatitis within 3 years after baseline. All three Cox models were then re-estimated within each of these increasingly restrictive cohorts.

All statistical analyses were performed using R software (version 4.2.2; R Foundation for Statistical Computing, Vienna, Austria). All statistical tests were two-sided, and a *p*-value <0.05 was considered statistically significant.

## Results

### Baseline characteristics

Table 1 presents the baseline characteristics of the study cohort. Among the 349,995 participants, 623 (0.18%) had chronic pancreatitis (CP). Compared to the non-CP group, the CP group was significantly older (58.95 ± 7.53 vs. 56.60 ± 8.08 years; *p* < 0.001) and included a higher proportion of males (66% vs. 46%; *p* < 0.001). The CP group also had a higher TDI score (−0.22 ± 3.54 vs. −1.37 ± 3.01; *p* < 0.001), indicating greater socioeconomic deprivation. Sleep duration was similar between the two groups (7.07 ± 1.46 vs. 7.15 ± 1.10 h; *p* = 0.053). No significant difference was observed in MET (2704.99 ± 2716.28 vs. 2644.92 ± 2656.03; *p* = 0.625). The CP group had a higher education score (20.46 ± 19.41 vs. 15.38 ± 16.00; *p* < 0.001). Ethnic distribution did not differ significantly (*p* = 0.777), with the majority being White in both groups (90.5% vs. 90.9%). A significantly higher percentage of CP patients were current smokers (25.5% vs. 10.2%; *p* < 0.001) and former drinkers (11.7% vs. 3.5%; *p* < 0.001). CP patients also had a significantly higher prevalence of pre-existing diabetes (17.8% vs. 5.1%; *p* < 0.001), a history of AP (8.2% vs. 0.2%; *p* < 0.001), and GSD (5.9% vs. 1.6%; *p* < 0.001). Metabolic and inflammatory profiles differed markedly. The CP group had a more adverse metabolic profile, including higher HbA1c, fasting glucose, triglycerides, cholesterol, low-density Lipoprotein (LDL), and WHR, alongside lower HDL cholesterol (all *p* < 0.001). Markers of systemic inflammation, such as CRP, monocyte count, and neutrophil count, were also elevated in the CP group (all *p* < 0.001). Consequently, the prevalence of all individual components of metabolic syndrome—central obesity, hypertension, dyslipidemia, hyperglycemia, and hypertriglyceridemia—was significantly higher in the CP group (all *p* < 0.001). Overall, metabolic syndrome was present in 34.3% of CP patients compared to 14.7% of non-CP participants (*p* < 0.001).

**Table 1:** Baseline characteristics of patients with chronic pancreatitis.

Characteristic	Non-CP	CP	*p*-value
*N*	349,372	623	
Age, mean (SD)	56.60 (8.08)	58.95 (7.53)	<0.001
Sex, mean (SD)	0.46 (0.50)	0.66 (0.47)	<0.001
TDI, mean (SD)	−1.37 (3.01)	−0.22 (3.54)	<0.001
Smoking status, *n* (%)			<0.001
Never	191,161 (54.7)	227 (36.4)	
Previous	122,463 (35.1)	237 (38.0)	
Current	35,767 (10.2)	159 (25.5)	
Sleep duration, mean (SD)	7.15 (1.10)	7.07 (1.46)	0.053
Alcohol drinker status, *n* (%)			<0.001
Never	15,187 (4.3)	28 (4.5)	
Previous	12,122 (3.5)	73 (11.7)	
Current	322,082 (92.2)	522 (83.8)	
MET, mean (SD)	2644.92 (2656.03)	2704.99 (2716.28)	0.625
Diabetes, *n* (%)			<0.001
No	330,558 (94.9)	508 (82.2)	
Yes	17,829 (5.1)	110 (17.8)	
Education score, mean (SD)	15.38 (16.00)	20.46 (19.41)	<0.001
Ethnity, *n* (%)			0.777
White	317,539 (90.9)	564 (90.5)	
Mixed	16,188 (4.6)	31 (5.0)	
Asian	13,574 (3.9)	26 (4.2)	
Black	2,090 (0.6)	2 (0.3)	
History of AP, *n* (%)			<0.001
No	348,832 (99.8)	572 (91.8)	
Yes	559 (0.2)	51 (8.2)	
History of GSD, *n* (%)			<0.001
No	343,891 (98.4)	586 (94.1)	
Yes	5,500 (1.6)	37 (5.9)	
BMI, mean (SD)	27.39 (4.73)	27.89 (5.04)	0.009
Body fat, mean (SD)	31.29 (8.52)	30.29±8.71	0.004
Wasit-to-hip ratio, mean (SD)	0.872 (0.22)	0.922 (0.31)	<0.001
HbA1c, mean (SD)	36.08 (6.65)	40.87 (12.19)	<0.001
HDL-c, mean (SD)	1.45 (0.38)	1.36 (0.40)	<0.001
LDL, mean (SD)	3.56 (0.87)	3.33 (0.93)	<0.001
Triglyceride, mean (SD)	1.74 (1.02)	2.00 (1.11)	<0.001
Glucose, mean (SD)	92.40 (21.81)	102.36 (38.59)	<0.001
Cholesterol, mean (SD)	5.70 (1.14)	5.38 (1.24)	<0.001
Calcium, mean (SD)	2.38 (0.09)	2.37 (0.11)	0.013
Total bilirubin, mean (SD)	9.15 (4.41)	9.15 (4.43)	0.990
CRP, mean (SD)	2.56 (4.27)	3.50 (5.25)	<0.001
Lymphocyte count, mean (SD)	1.97 (1.16)	2.01 (0.72)	0.380
Lymphocyte percentage, mean (SD)	28.95 (7.49)	27.35 ± 8.06	<0.001
Monocyte count, mean (SD)	0.48 (0.29)	0.53 (0.27)	<0.001
Neutrophill count, mean (SD)	4.22 (1.41)	4.72 (1.72)	<0.001
Neutrophill percentage, mean (SD)	60.84 (8.52)	62.13 (9.43)	<0.001
Platelet count, mean (SD)	252.01 (59.61)	248.97 (69.11)	0.211
White blood cell, mean (SD)	6.89 (2.13)	7.50 (2.12)	<0.001
Hct, mean (SD)	41.16 (3.55)	41.51 (3.81)	0.016
Hb, mean (SD)	14.19 (1.24)	14.32 (1.32)	0.011
Energy intake [mean (SD)]	8641.21 (2541.70)	9117.16 (2788.26)	0.007
Central obesity, *n* (%)			<0.001
No	304,952 (87.3)	477 (76.6)	
Yes	44,439 (12.7)	146 (23.4)	
Hypertension, *n* (%)			<0.001
No	109,967 (31.5)	150 (24.1)	
Yes	239,424 (68.5)	473 (75.9)	
Dyslipidemia, *n* (%)			<0.001
No	311,056 (89.0)	468 (75.1)	
Yes	38,335 (11.0)	155 (24.9)	
Hyperglycemia, *n* (%)			<0.001
No	318,378 (91.1)	456 (73.2)	
Yes	31,013 (8.9)	167 (26.8)	
Hypertriglyceridemia, *n* (%)			<0.001
No	210,032 (60.1)	303 (48.6)	
Yes	139,359 (39.9)	320 (51.4)	
MetS, *n* (%)			<0.001
No	298,070 (85.3)	409 (65.7)	
Yes	51,321 (14.7)	214 (34.3)	

CP, Chronic pancreatitis; BMI, body mass index; MET, metabolic equivalent task; TDI, Townsend deprivation index; AP, acute pancreatitis; GSD, gallstone disease; HbA1c, Hemoglobin A1c; HDL-c, High-Density Lipoprotein cholesterol; LDL, Low-Density Lipoprotein; CRP, C-reactive Protein; Hct, Hematocrit; Hb, Hemoglobin; MetS, metabolic syndrome; SD, Standard.

Table 2 presents the characteristics of the study population stratified by the cumulative number of MetS traits. Among 349,995 participants, 72,249 (20.6%) had zero MetS traits, and 51,531 (14.7%) had three or more traits, fulfilling the criteria for MetS. A clear positive gradient was observed between the number of MetS traits and age, BMI, waist circumference, and waist-to-hip ratio (all *p* < 0.001). Correspondingly, metabolic parameters worsened progressively: HbA1c, fasting glucose, and triglyceride levels increased, while HDL cholesterol levels decreased (all *p* < 0.001). The prevalence of hypertension and hyperglycemia rose steeply with the number of MetS traits. All participants with five traits had hypertension and hyperglycemia, whereas none with zero traits had these conditions. The proportion of males increased across trait categories, from 22.6% in the zero-trait group to 100% in groups with four or five traits (*p* < 0.001).

**Table 2:** Demographic characteristics of study population by number of metabolic syndrome traits.

Characteristics	*N*	0 *N* = 72,249	1 *N* = 132,802	2 *N* = 93,413	3 *N* = 35,882	4 *N* = 11,726	5 *N* = 3,923	*p*-value
Hypertension, *n* (%)	349,995							<0.001
No		72,249 (100.00%)	31,032 (23.37%)	5,760 (6.17%)	888 (2.47%)	184 (1.57%)	0 (0.00%)	
Yes		0 (0.00%)	101,770 (76.63%)	87,653 (93.83%)	34,994 (97.53%)	11,542 (98.43%)	3,923 (100.00%)	
Hyperglycemia, *n* (%)	349,995							<0.001
No		72,249 (100.00%)	130,627 (98.36%)	85,563 (91.60%)	25,104 (69.96%)	5,280 (45.03%)	0 (0.00%)	
Yes		0 (0.00%)	2,175 (1.64%)	7,850 (8.40%)	10,778 (30.04%)	6,446 (54.97%)	3,923 (100.00%)	
Sex, *n* (%)	349,995							<0.001
No		55,931 (77.41%)	84,913 (63.94%)	41,913 (44.87%)	5,625 (15.68%)	0 (0.00%)	0 (0.00%)	
Yes		16,318 (22.59%)	47,889 (36.06%)	51,500 (55.13%)	30,257 (84.32%)	11,726 (100.00%)	3,923 (100.00%)	
Age, mean ± SD	349,995	52.51 ± 7.90	56.72 ± 7.92	58.11 ± 7.66	58.83 ± 7.53	60.29 ± 6.76	60.49 ± 6.48	<0.001
BMI, mean ± SD	349,995	24.82 ± 3.74	26.52 ± 4.25	28.46 ± 4.47	30.73 ± 4.54	31.94 ± 4.26	34.21 ± 4.61	<0.001
WC, mean ± SD	349,995	80.96 ± 10.12	86.73 ± 10.90	94.18 ± 10.97	103.91 ± 10.97	108.99 ± 10.03	115.01 ± 10.15	<0.001
WTHR, mean ± SD	349,995	0.81 ± 0.07	0.85 ± 0.08	0.90 ± 0.07	0.96 ± 0.07	1.00 ± 0.06	1.02 ± 0.06	<0.001
Body fat, mean ± SD	349,995	30.43 ± 8.36	31.53 ± 9.03	31.82 ± 8.86	30.85 ± 6.95	30.58 ± 4.56	32.52 ± 4.40	<0.001
HbA1c, mean ± SD	331,126	33.88 ± 3.34	34.83 ± 4.05	36.35 ± 6.20	39.88 ± 9.90	44.31 ± 11.91	53.29 ± 13.13	<0.001
HDL-c, mean ± SD	349,995	1.61 ± 0.37	1.56 ± 0.39	1.33 ± 0.31	1.19 ± 0.26	1.11 ± 0.24	1.03 ± 0.23	<0.001
Glu, mean ± SD	349,995	86.87 ± 10.83	89.95 ± 13.03	92.82 ± 19.87	100.43 ± 34.69	111.47 ± 45.35	137.87 ± 60.02	<0.001
TG, mean ± SD	349,995	1.06 ± 0.32	1.41 ± 0.71	2.27 ± 1.04	2.55 ± 1.21	2.62 ± 1.25	2.96 ± 1.26	<0.001
LDL, mean ± SD	349,995	3.38 ± 0.75	3.62 ± 0.81	3.77 ± 0.93	3.43 ± 0.93	2.96 ± 0.84	2.68 ± 0.67	<0.001

BMI, body mass index; WC, waist circumference; WTHR, waist-to-hip ratio; HbA1c, glycated hemoglobin A1c; HDL-c, high-density lipoprotein; LDL, low-density lipoprotein; Glu, glucose; TG, Triglyceride.

### The associations between metabolic syndrome and the risk of CP

Cox regression results for the associations between metabolic syndrome components and incident chronic pancreatitis are shown in Table 3. In the crude model (Model 1), all individual MetS components and overall MetS were significantly associated with higher CP risk. The strongest association was with hyperglycemia (HR 3.93, 95% CI 3.29–4.69). A positive dose–response relationship was observed for WHR, while higher body fat percentage was inversely associated with CP risk. After adjusting for demographic, lifestyle, and clinical factors (Model 2), hyperglycemia remained strongly associated (HR 2.73, 95% CI 2.25–3.30), followed by dyslipidemia, hypertriglyceridemia, and central obesity. Hypertension was no longer significant. The association for body fat percentage reversed direction, showing increased risk in higher quartiles. WHR maintained a strong positive trend (*p* for trend <0.001). MetS was associated with a twofold increased risk (HR 2.09, 95% CI 1.71–2.56). Further adjustment for inflammatory and hematological biomarkers (Model 3) yielded consistent results. Hyperglycemia (HR 2.59, 95% CI 2.12–3.16) and elevated WHR (Q4 vs. Q1: HR 2.78, 95% CI 1.91–4.05) remained the strongest independent predictors. MetS retained a significant association with CP (HR 2.02, 95% CI 1.64–2.49).

**Table 3:** The association between metabolic syndrome and the risk of chronic pancreatitis.

Type	Model 1		Model 2		Model 3	
	HR (95% CI)	*p*-value	HR (95% CI)	*p*-value	HR (95% CI)	*p*-value
Hyperglycemia						
No	Reference		Reference		Reference	
Yes	3.928 (3.29–4.69)	<0.001	2.727 (2.251–3.303)	<0.001	2.588 (2.12–3.158)	<0.001
Hypertension						
No	Reference		Reference		Reference	
Yes	1.474 (1.23–1.77)	<0.001	1.046 (0.858–1.274)	0.6568	1.048 (0.86–1.28)	0.6489
Central obesity						
No	Reference		Reference		Reference	
Yes	2.164 (1.80–2.60)	<0.001	1.367 (1.075–1.739)	0.0109	1.344 (1.05–1.72)	0.018
Dyslipidemia						
No	Reference		Reference		Reference	
Yes	2.808 (2.34–3.37)	<0.001	1.496 (1.215–1.842)	<0.001	1.393 (1.13–1.73)	0.0023
Hypertriglyceridemia						
No	Reference		Reference		Reference	
Yes	1.605 (1.37–1.88)	<0.001	1.247 (1.058–1.47)	0.0083	1.311 (1.11–1.55)	0.0018
Body fat						
Q1	Reference		Reference		Reference	
Q2	1.017 (0.83–1.25)	0.8752	1.212 (0.961–1.527)	0.1037	1.297 (1.02–1.64)	0.0313
Q3	0.778 (0.62–0.97)	0.028	1.549 (1.135–2.114)	0.0058	1.605 (1.17–2.21)	0.0037
Q4	0.755 (0.60–0.94)	0.0148	2.368 (1.502–3.733)	<0.001	2.568 (1.61–4.098)	<0.001
*p* for trend		<0.001		<0.001		<0.001
Waist-to-hip ratio						
Q1	Reference		Reference		Reference	
Q2	1.684 (1.23–2.29)	0.001	1.44 (1.043–1.988)	0.0266	1.438 (1.03–1.99)	0.0305
Q3	2.858 (2.14–3.80)	<0.001	2.053 (1.47–2.869)	<0.001	2.048 (1.45–2.88)	<0.001
Q4	4.476 (3.4–5.87)	<0.001	2.788 (1.932–4.023)	<0.001	2.783 (1.91–4.05)	<0.001
*p* for trend		<0.001		<0.001		<0.001
MetS						
No	Reference		Reference		Reference	
Yes	3.151 (2.67–3.72)	<0.001	2.094 (1.71–2.564)	<0.001	2.022 (1.64–2.49)	<0.001

Model 1 has no variables adjusted; Model 2 adjusted age, sex, TDI, smoking status, alcohol drinker status, education score, history of AP, history of GSD, BMI, and energy intake; Model 3 further adjusted with calcium, CRP, lymphocyte percentage, monocyte count, neutrophil count and percentage, white blood cell count, Hct, and Hb.

The association between the cumulative number of MetS traits and CP risk demonstrated a clear dose–response pattern (Table 4). In the unadjusted model, risk increased progressively with trait accumulation, peaking at four traits (HR 5.87, 95% CI 4.23–8.17). This graded relationship persisted after full adjustment (*p* for trend <0.001). Compared to individuals with zero traits, those with two, three, four, and five traits had adjusted hazard ratios of 1.66 (95% CI 1.22–2.25), 2.50 (95% CI 1.76–3.55), 3.18 (95% CI 2.10–4.82), and 2.14 (95% CI 1.15–4.00), respectively.

**Table 4:** Hazard ratios (HR) (95% CI) for the comprehensive analysis of chronic pancreatitis risk associated with the clustering of metabolic syndrome.

No. of metabolic syndrome traits	CP (%)	HR (95%CI)	*p* value
Model 1			
0	72,249 (0.104)	Reference	
1	132,802 (0.111)	1.085 (0.822–1.433)	0.563
2	93,413 (0.199)	1.962 (1.500–2.565)	<0.001
3	35,882 (0.365)	3.665 (2.759–4.868)	<0.001
4	11,726 (0.571)	5.874 (4.225–8.167)	<0.001
5	3,923 (0.408)	4.337 (2.528–7.441)	<0.001
*P* for trend		1.570 (1.476–1.670)	<0.001
Model 2			
0	72,249 (0.104)	Reference	
1	132,802 (0.111)	0.929 (0.697–1.238)	0.617
2	93,413 (0.199)	1.502 (1.119–2.016)	0.007
3	35,882 (0.365)	2.422 (1.727–3.397)	<0.001
4	11,726 (0.571)	3.262 (2.187–4.866)	<0.001
5	3,923 (0.408)	2.412 (1.325–4.391)	0.004
*p* for trend		1.371 (1.263–1.489)	<0.001
Model 3			
0	72,249 (0.104)	Reference	
1	132,802 (0.111)	0.978 (0.727–1.316)	0.882
2	93,413 (0.199)	1.656 (1.219–2.251)	0.0013
3	35,882 (0.365)	2.501 (1.761–3.554)	<0.001
4	11,726 (0.571)	3.179 (2.098–4.817)	<0.001
5	3,923 (0.408)	2.141 (1.147–3.997)	0.017
*p* for trend		1.345 (1.234–1.465)	<0.001

Model 1 has no variables adjusted; Model 2 adjusted age, sex, TDI, smoking status, alcohol drinker status, education score, history of AP, history of GSD, BMI, and energy intake; Model 3 further adjusted with calcium, CRP, lymphocyte percentage, monocyte count, neutrophil count and percentage, white blood cell count, Hct, and Hb.

### Nonlinear analysis of metabolic syndrome and CP

**Figure 2:**
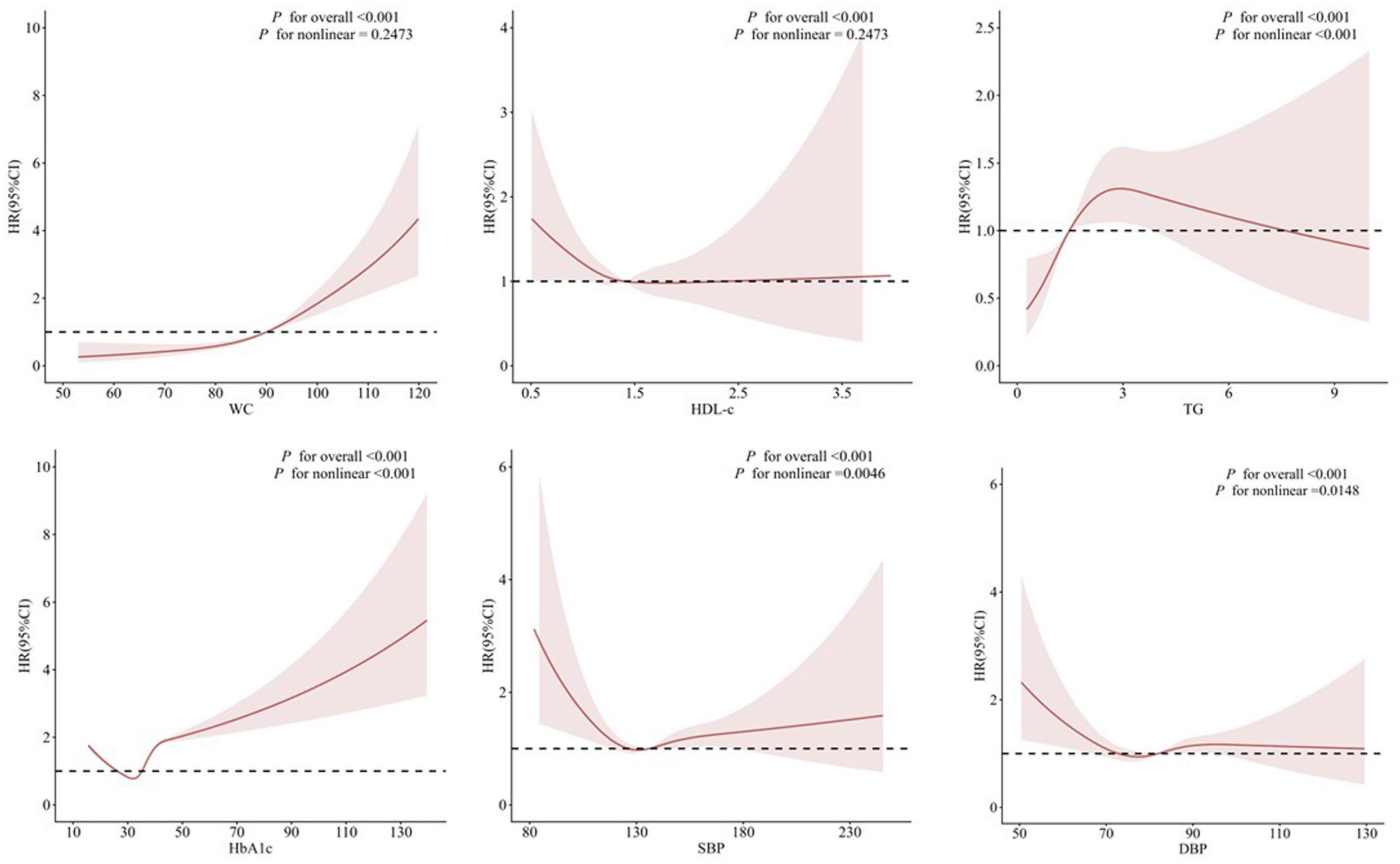
Nonlinear association between MetS components and the risk of chronic pancreatitis.

As shown in Figure 2, dose–response relationships between individual components of metabolic syndrome and the risk of CP were assessed using RCS models. The overall association was statistically significant for all components (*p* for overall <0.001). The shapes of the dose–response curves varied between components. WC, HbA1c, and SBP exhibited essentially monotonic positive associations, with risk increasing progressively across their respective ranges. In contrast, DBP demonstrated a J-shaped curve, with the lowest risk observed around 70–80 mmHg and increased risk at both lower and higher values. HDL-c and TG displayed U-shaped associations. For HDL-c, the risk was elevated at both low and high levels, with the lowest risk observed in the middle of the range. Similarly, TG showed a modest inverse association at lower levels, but the risk increased substantially at higher concentrations. Tests for nonlinearity were significant for all components except WC, indicating that a linear model would not adequately capture the complex associations for most MetS components with CP risk.

### Prognostic value of metabolic syndrome and CP

**Figure 3:**
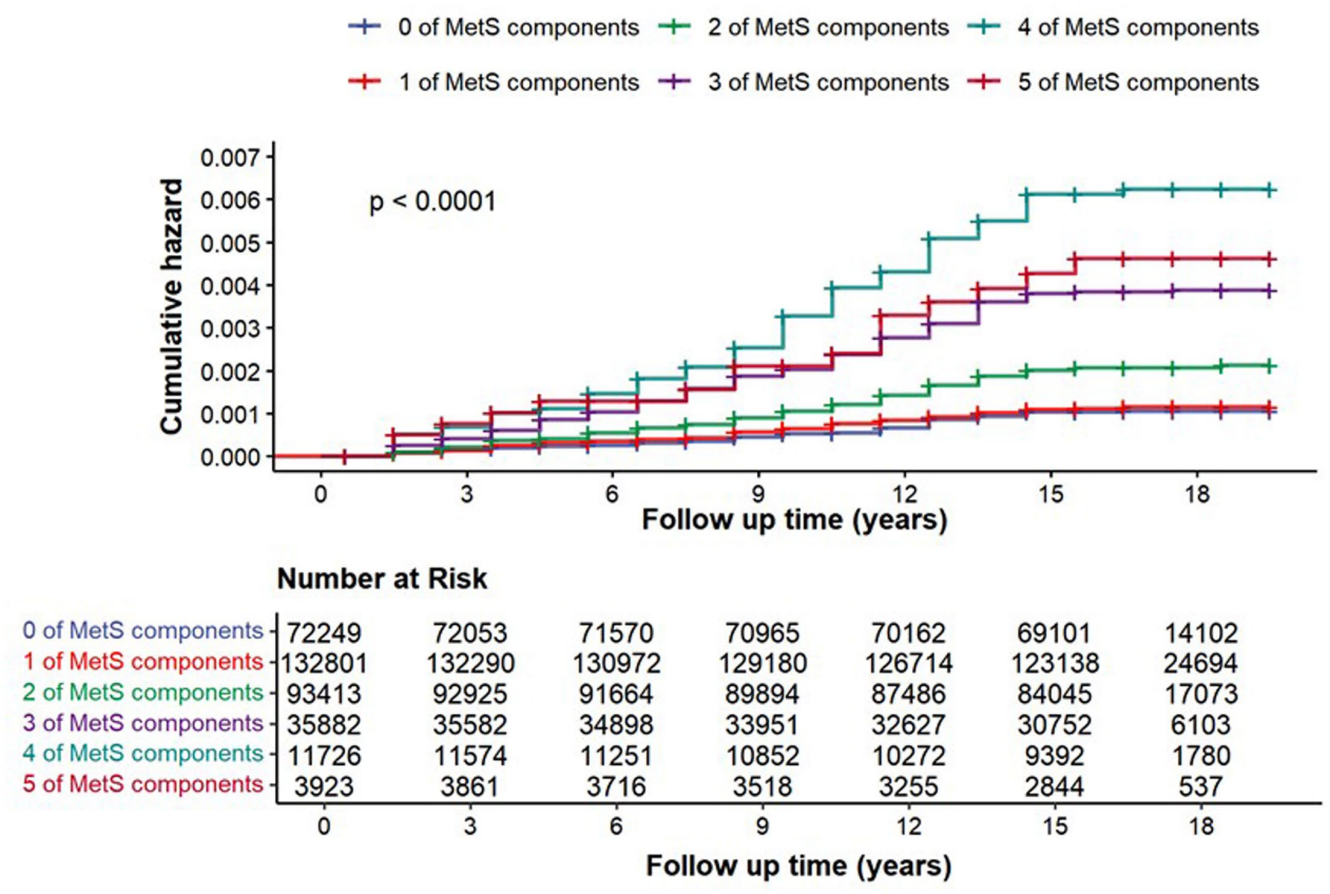
Kaplan–Meier curves for cumulative incidence of chronic pancreatitis stratified by MetS components.

Figure 3 illustrates the Kaplan–Meier cumulative incidence curves for chronic pancreatitis, stratified according to the number of present MetS components (0–5). The survival curves exhibited significant separation across strata (log-rank *p* < 0.0001). A clear dose–response relationship was evident, whereby a greater number of MetS components was associated with progressively higher cumulative incidence of chronic pancreatitis over time. Specifically, individuals with zero MetS components showed the lowest cumulative risk, whereas those with four or five components displayed the earliest and steepest rise in incidence, maintaining the highest risk throughout the 18-year follow-up. The accompanying risk table (covering 0–18 years) details the number of participants remaining under observation in each stratum at 3-year intervals, reflecting gradual attrition during follow-up.

### Subgroup analysis

**Figure 4:**
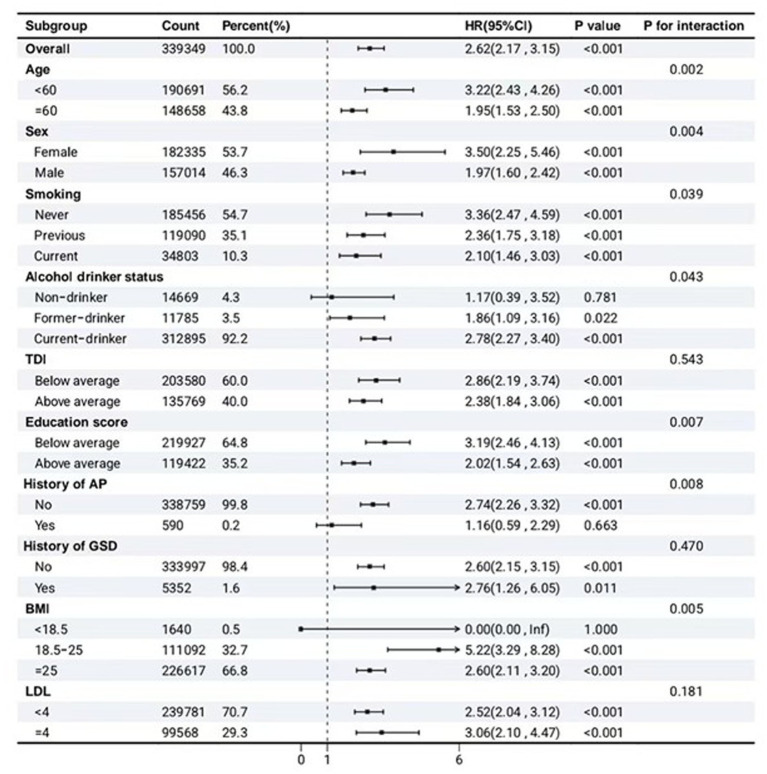
Subgroup analysis of the association between MetS and the risk of chronic pancreatitis. TDI, Townsend deprivation index; AP, acute pancreatitis; GSD, gallstone disease; BMI, body mass index.

In the subgroup analysis (Figure 4), MetS was consistently associated with an elevated risk of chronic pancreatitis across most population strata. Overall, the HR was 2.62 (95% CI: 2.17–3.15, *p* < 0.001). Significant effect modifications were observed for age, sex, smoking status, alcohol consumption, BMI, and educational level (*p* for interaction <0.05 for all). Notably, stronger associations were found in younger participants (<60 years, HR = 3.22, 95% CI: 2.43–4.26) compared to older ones (≥60 years, HR = 1.95, 95% CI: 1.53–2.50), and in females (HR = 3.50, 95% CI: 2.25–5.46) compared to males (HR = 1.97, 95% CI: 1.60–2.42). Never smokers showed a higher risk (HR = 3.36, 95% CI: 2.47–4.59) than current or former smokers. Alcohol drinkers exhibited a significantly elevated risk (HR = 2.78, 95% CI: 2.27–3.40), whereas non-drinkers did not (HR = 1.17, 95% CI: 0.39–3.52, *p* = 0.781). Interestingly, individuals with a BMI between 18.5 and 25 kg/m^2^ had the highest risk (HR = 5.22, 95% CI: 3.29–8.28), while no significant interaction was observed for TDI or LDL cholesterol levels (*p* for interaction >0.05).

### Mediation analysis

**Figure 5:**
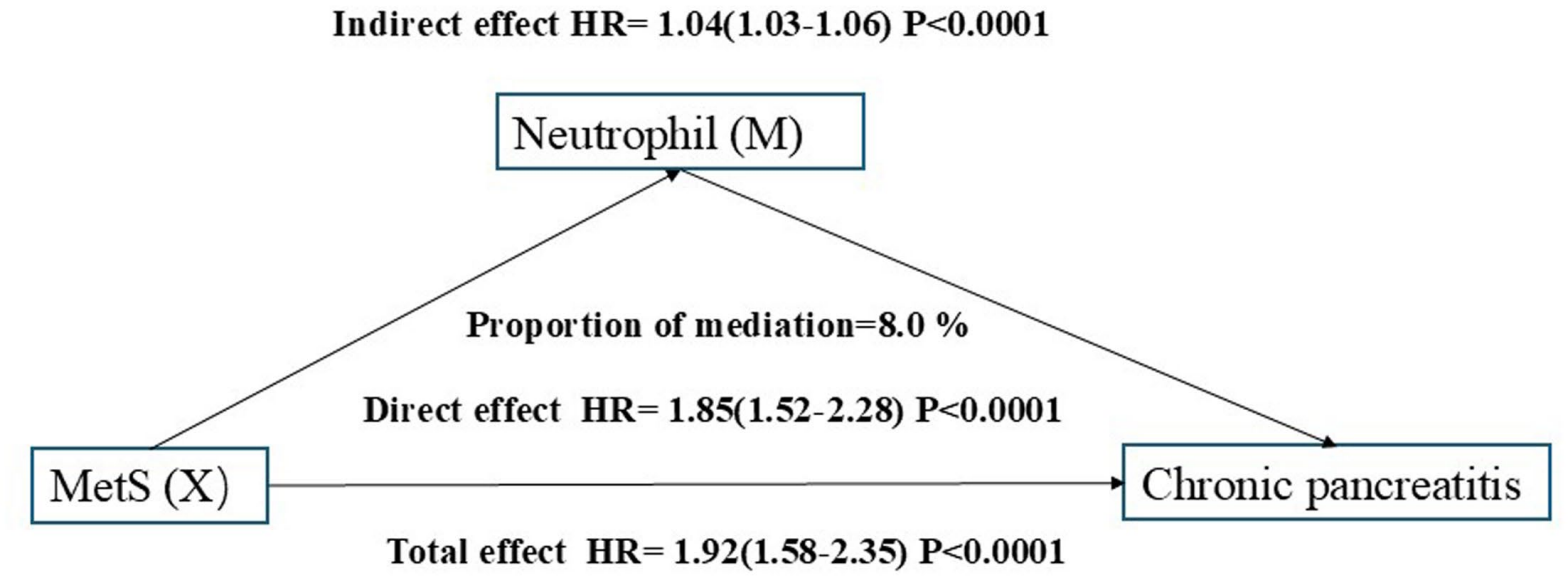
Mediation role of neutrophil in the associati on between MetS and chronic pancreatitis risk.

**Figure 6:**
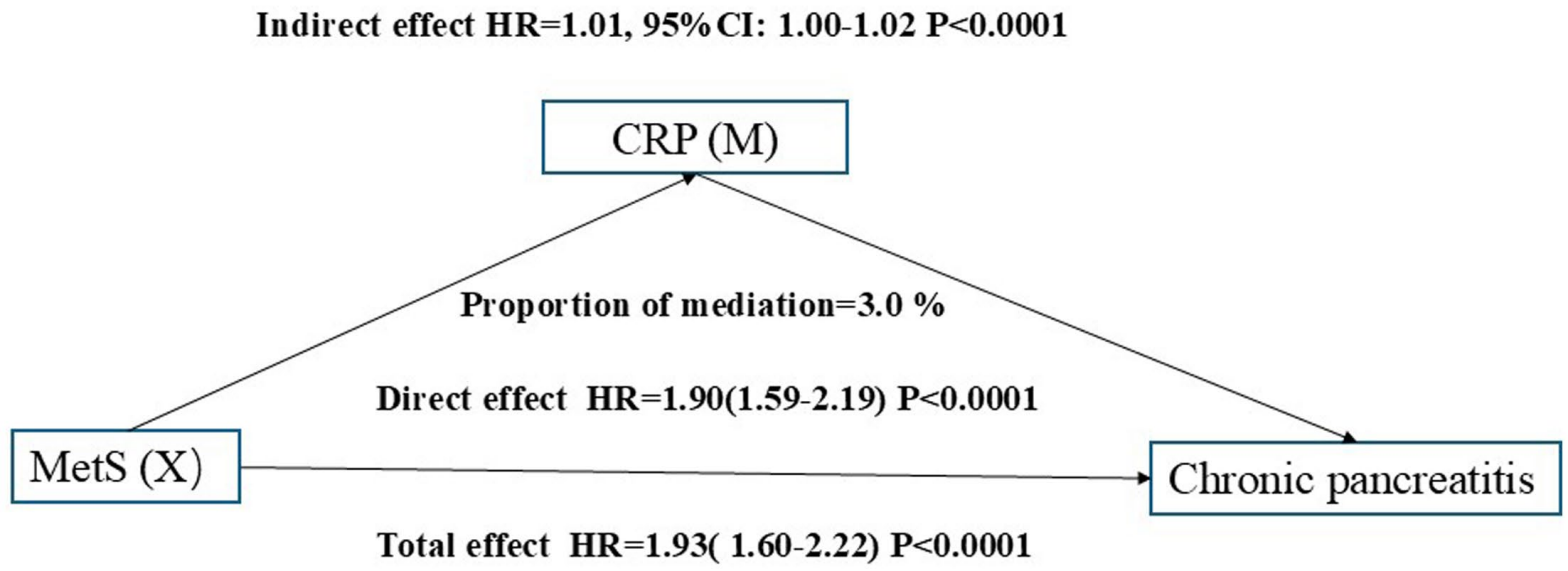
Mediation role of C-Reactive Protein (CRP) in the association between MatS and chronic pancreatitis risk.

A combined mediation analysis was conducted to evaluate the roles of systemic inflammatory markers in the association between MetS and chronic pancreatitis risk, adjusting for age, sex, TDI, smoking status, alcohol drinker status, education score, history of AP, history of GSD, BMI, energy intake, calcium, Hct, and Hb. Inflammatory markers were not included as covariates because they are highly correlated with the mediators. The total effect of MetS on chronic pancreatitis was significant in both models, with HRs of 1.92 (95% CI: 1.58–2.35) for neutrophils (Figure 5) and 1.93 (95% CI: 1.60–2.22) for CRP (Figure 6), both *p* < 0.0001. After adjustment, the direct effects remained strong and significant (HR = 1.85, 95% CI: 1.52–2.28 for neutrophils; HR = 1.90, 95% CI: 1.59–2.19 for CRP). The indirect effects mediated through neutrophils (HR = 1.04, 95% CI: 1.03–1.06) and CRP (HR = 1.01, 95% CI: 1.00–1.02) were both statistically significant (*p* < 0.0001). Neutrophils accounted for approximately 8.0% of the total effect, whereas CRP explained about 3.0% of the mediation.

### Sensitivity analysis

To assess the robustness of our primary findings, we performed multiple sensitivity analyses. First, after multiple imputation for missing data, MetS remained significantly associated with chronic pancreatitis risk in all models: HR = 3.151 (95% CI: 2.671–3.717) in Model 1, HR = 2.128 (1.738–2.605) in Model 2, and HR = 2.056 (1.678–2.520) in Model 3 (all *p* < 0.001). A dose–response relationship was also preserved across the number of MetS traits (*p* for trend <0.001) (Supplementary Table S1). Second, after excluding participants who died or were lost to follow-up, the association persisted, with adjusted HRs of 1.730 (1.324–2.261) in Model 2 and 1.671 (1.270–2.199) in Model 3 (Supplementary Table S2). Third, further exclusion of incident cases diagnosed within 3 years of follow-up yielded consistent results: HR = 2.236 (1.783–2.803) in Model 2 and HR = 2.184 (1.732–2.754) in Model 3 (Supplementary Table S3). In all analyses, hyperglycemia and dyslipidemia were consistently the strongest individual components associated with chronic pancreatitis risk, whereas hypertension and central obesity became non-significant after full adjustment. These sensitivity analyses confirm the stability of the observed association between MetS and chronic pancreatitis risk across different methodological assumptions and follow-up scenarios.

## Discussion

The present study provides a comprehensive, population-based analysis of the association between metabolic syndrome and the risk of incident chronic pancreatitis, leveraging extensive data from the UK Biobank cohort. Our findings demonstrate that metabolic syndrome is an independent and significant risk factor for chronic pancreatitis, with a clear dose–response relationship observed between the number of metabolic syndrome components and disease risk. These results expand the understanding of the metabolic determinants of chronic pancreatitis beyond traditional factors such as alcohol and smoking.

Existing literature consistently indicates differential roles for individual metabolic components in CP pathogenesis. For obesity, particularly central adiposity, meta-analyses confirm a significant positive association, with a pooled relative risk of approximately 1.40 (95% CI: 1.18–1.66) for obese individuals, primarily mediated through chronic inflammation and adipokine dysregulation (27). Regarding diabetes, prospective cohort studies have identified type 2 diabetes as an independent risk factor, reporting hazard ratios around 1.82 (95% CI: 1.40–2.36), with proposed mechanisms involving hyperglycemia-induced oxidative stress and pancreatic microangiopathy (28). For dyslipidemia, severe hypertriglyceridemia is a well-established, direct risk factor for both acute and chronic pancreatitis, with population studies indicating a more than twofold increased risk (HR ≈ 2.54) for moderate-to-severe elevations (29); however, associations with other lipid parameters like HDL-C or LDL-C remain inconclusive. In contrast, the evidence for hypertension is notably weaker, with most large-scale studies—such as a Nordic cohort following over 64,000 participants—finding no independent association after adjustment for confounders (HR ≈ 1.05, 95% CI: 0.82–1.34), suggesting its presence in CP patients may largely reflect comorbidity rather than causality (30). Collectively, these findings underscore that obesity, diabetes, and hypertriglyceridemia are the core metabolic drivers of CP risk, while highlighting the necessity to evaluate their synergistic effect as part of the metabolic syndrome cluster.

The findings of the present study align closely with and significantly extend the existing literature summarized above. Our results corroborate the central role of obesity, diabetes, and hypertriglyceridemia as core metabolic drivers of CP risk. Specifically, we identified hyperglycemia (HR in fully adjusted model = 2.59) and elevated waist-to-hip ratio (Q4 vs. Q1 HR = 2.78) as the strongest independent predictors, which is consistent with prior meta-analyses and cohort studies highlighting diabetes and central adiposity. Furthermore, our data confirm the significant risk associated with hypertriglyceridemia, reinforcing its established etiological role.

However, our study provides critical advancements. First, we demonstrate that when these components cluster as metabolic syndrome, the combined risk is substantial (HR = 2.02) and exhibits a clear dose–response relationship, directly addressing the literature’s call to evaluate their synergistic effect. Second, we offer novel mechanistic insights by quantifying the mediating role of systemic inflammation—specifically through neutrophils (≈8% mediation) and CRP (≈3% mediation)—thereby providing epidemiological support for the pathophysiological pathways previously proposed in experimental studies, where chronic inflammation is understood to drive pancreatic injury through multiple mechanisms: sustained activation of pancreatic stellate cells leading to fibrosis (31), promotion of acinar cell injury and death, and the creation of a pro-fibrotic microenvironment through cytokine and chemokine release (32). Third, our findings clarify the ambiguous role of hypertension: similar to the large Nordic cohort study, hypertension was not independently associated with CP risk in our fully adjusted models, confirming that its apparent link is likely attributable to confounding by other metabolic factors.

The Kaplan–Meier curves demonstrate a clear graded relationship between the number of metabolic syndrome components and the cumulative incidence of chronic pancreatitis. With each additional component, the risk increases progressively, and individuals with four or five components exhibit the earliest and steepest rise in incidence. The significant separation of the curves throughout follow-up confirms a strong, sustained dose–response effect, underscoring the importance of cumulative metabolic burden in chronic pancreatitis development.

Notably, our study revealed significant heterogeneity in risk across population subgroups. The stronger association observed in younger individuals (<60 years) and females suggests that the metabolic susceptibility to pancreatic injury may vary across the lifespan and by sex, possibly due to hormonal influences or differences in body composition (33). The unexpected finding of the highest risk in individuals with a normal BMI (18.5–25 kg/m^2^) warrants further investigation, as it may indicate that metabolic dysregulation in the absence of overall obesity carries a distinct risk profile, or it may reflect the limitations of BMI as a measure of adiposity compared to waist-to-hip ratio.

The robust dose–response relationship, evident in both linear and non-linear analyses, strengthens the argument for a causal link. The U-shaped or J-shaped associations observed for some metabolic components (e.g., HDL-c, TG, DBP) suggest that both extremes of these physiological measures may be detrimental, implying that optimal pancreatic health may exist within a narrow homeostatic range. The consistency of our primary findings across multiple sensitivity analyses—including multiple imputation, exclusion of early cases, and accounting for competing risks—supports the robustness of the conclusions and mitigates concerns related to missing data, reverse causality, and selection bias.

While alcohol remains the dominant etiological factor for chronic pancreatitis in Western populations (34), this study provides the first comprehensive, large-scale prospective evidence establishing metabolic syndrome as an independent and significant risk factor for chronic pancreatitis. Our results position metabolic syndrome as an important and modifiable contributor to disease burden. This has significant clinical and public health implications, particularly given the rising global prevalence of obesity and metabolic disorders. Future research should focus on elucidating the molecular mechanisms linking specific metabolic traits to pancreatic injury, evaluating whether interventions targeting metabolic syndrome (e.g., weight loss, glycemic control, lipid management) can reduce the incidence or progression of chronic pancreatitis, and exploring potential genetic interactions that may modulate individual susceptibility.

This study has several notable strengths, including the large, prospective, and well-phenotyped cohort, the use of objective clinical and biochemical measurements for defining metabolic syndrome, comprehensive adjustment for a wide range of potential confounders, and the application of multiple advanced statistical methods to assess association patterns, mediation, and robustness. The exclusion of 73,152 participants (17.3%) due to missing covariate data may introduce selection bias and somewhat limit generalizability. However, the remaining sample remains large (*n* = 349,995), and missingness was spread across multiple variables, suggesting that the impact on the overall findings is likely limited.

However, certain limitations should be acknowledged. First, despite extensive adjustment, residual confounding from unmeasured or imperfectly measured factors (e.g., detailed dietary patterns, genetic predisposition not captured by basic covariates) cannot be ruled out. Second, the diagnosis of chronic pancreatitis relied on hospital admission and death registry codes, which may under-ascertain milder or outpatient-managed cases. Third, the UK Biobank cohort, while large, is not fully representative of the general population, as participants tend to be healthier and from less deprived backgrounds, which may affect the generalizability of risk estimates (35). Finally, the observational nature of the study precludes definitive causal inference.

## Conclusion

In conclusion, this large prospective cohort study demonstrates that metabolic syndrome and its individual components, particularly hyperglycemia and central adiposity, are independently associated with an increased risk of chronic pancreatitis. The association exhibits a dose–response pattern, is partially mediated by systemic inflammation, and remains robust across multiple sensitivity analyses. These findings underscore the importance of metabolic health in pancreatic disease prevention and suggest that screening for and management of metabolic syndrome may play a role in mitigating the risk of chronic pancreatitis, alongside traditional measures such as limiting alcohol consumption and smoking cessation.

## Data Availability

The original contributions presented in the study are included in the article/Supplementary material, further inquiries can be directed to the corresponding authors.
