# Supplementary material for: Metabolic syndrome and risk of incident chronic pancreatitis: a prospective cohort study
Source: Front Nutr. 2026 Apr 29;13:1817098. doi: 10.3389/fnut.2026.1817098 (PMC13169131; doi:10.3389/fnut.2026.1817098)
Supplement: Supplementary file 1 [file Table_1.DOCX]

| Supplementary Table 1. The association between metabolic syndrome and the risk of chronic pancreatitis (Multiple Imputation) | | | | | | |
| --- | --- | --- | --- | --- | --- | --- |
|  | **Model 1** | | **Model 2** | | **Model 3** | |
| Type | **HR (95% CI)** | ***P*-Value** | **HR (95% CI)** | **P-Value** | **HR (95% CI)** | ***P*-Value** |
| Hyperglycemia |  |  |  |  |  |  |
| No | Reference |  | Reference |  | Reference |  |
| Yes | 3.928 (3.290-4.690) | <0.001 | 2.717 (2.243-3.292) | <0.001 | 2.565 (2.111-3.117) | <0.001 |
| Hypertension |  |  |  |  |  |  |
| No | Reference |  | Reference |  | Reference |  |
| Yes | 1.474 (1.227-1.772) | <0.001 | 1.052 (0.864-1.28) | 0.615 | 1.067 (0.875-1.3) | 0.524 |
| Central obesity |  |  |  |  |  |  |
| No | Reference |  | Reference |  | Reference |  |
| Yes | 2.164 (1.797-2.604) | <0.001 | 1.378 (1.083-1.754) | 0.009 | 1.364 (1.072-1.736) | 0.012 |
| Dyslipidemia |  |  |  |  |  |  |
| No | Reference |  | Reference |  | Reference |  |
| Yes | 2.808 (2.341-3.367) | <0.001 | 1.522 (1.236-1.874) | <0.001 | 1.434 (1.163-1.769) | 0.001 |
| Hypertriglyceridemia |  |  |  |  |  |  |
| No | Reference |  | Reference |  | Reference |  |
| Yes | 1.605 (1.372-1.878) | <0.001 | 1.246 (1.057-1.468) | 0.009 | 1.266 (1.072-1.494) | 0.005 |
| No. of Metabolic Syndrome traits |  |  |  |  |  |  |
| 0 | Reference |  | Reference |  | Reference |  |
| 1 | 1.085 (0.822-1.433) | 0.563 | 0.914 (0.687-1.216) | 0.537 | 0.946 (0.71-1.259) | 0.702 |
| 2 | 1.962 (1.500-2.565) | <0.001 | 1.484 (1.107-1.989) | 0.008 | 1.522 (1.132-2.045) | 0.005 |
| 3 | 3.665 (2.759-4.868) | <0.001 | 2.419 (1.727-3.389) | <0.001 | 2.436 (1.735-3.42) | <0.001 |
| 4 | 5.874 (4.225-8.167) | <0.001 | 3.311 (2.224-4.928) | <0.001 | 3.257 (2.183-4.861) | <0.001 |
| 5 | 4.337 (2.528-7.441) | <0.001 | 2.400 (1.319-4.367) | 0.004 | 2.196 (1.203-4.008) | 0.010 |
| P for trend | 1.570 (1.476-1.670) | <0.001 | 1.377 (1.268-1.495) | <0.001 | 1.356 (1.249-1.473) | <0.001 |
| MetS |  |  |  |  |  |  |
| No | Reference |  | Reference |  | Reference |  |
| Yes | 3.151 (2.671-3.717) | <0.001 | 2.128 (1.738-2.605) | <0.001 | 2.056 (1.678-2.52) | <0.001 |

Model 1 has no variables adjusted; Model 2 adjusted age, sex, TDI, smoking status, alcohol drinker status, education score, history of AP, history of GSD, BMI, and energy intake; Model 3 further adjusted with calcium, CRP, lymphocyte percentage, monocyte count, neutrophil count and percentage, white blood cell count, Hct, and Hb.

| Supplementary Table 2. The association between metabolic syndrome and the risk of chronic pancreatitis (Excluding Deaths and Loss to Follow-up) | | | | | | |
| --- | --- | --- | --- | --- | --- | --- |
|  | **Model 1** | | **Model 2** | | **Model 3** | |
| Type | **HR (95% CI)** | ***P*-Value** | **HR (95% CI)** | **P-Value** | **HR (95% CI)** | ***P*-Value** |
| Hyperglycemia |  |  |  |  |  |  |
| No | Reference |  | Reference |  | Reference |  |
| Yes | 3.134 (2.446-4.016) | <0.001 | 2.152 (1.649-2.809) | <0.001 | 2.105 (1.600-2.769) | <0.001 |
| Hypertension |  |  |  |  |  |  |
| No | Reference |  | Reference |  | Reference |  |
| Yes | 1.395 (1.109-1.756) | 0.005 | 1.048 (0.819-1.342) | 0.708 | 0.984 (0.766-1.264) | 0.900 |
| Central obesity |  |  |  |  |  |  |
| No | Reference |  | Reference |  | Reference |  |
| Yes | 2.03 (1.586-2.598) | <0.001 | 1.233 (0.900-1.689) | 0.193 | 1.199 (0.869-1.655) | 0.269 |
| Dyslipidemia |  |  |  |  |  |  |
| No | Reference |  | Reference |  | Reference |  |
| Yes | 2.645 (2.073-3.374) | <0.001 | 1.554 (1.176-2.053) | 0.002 | 1.519 (1.141-2.022) | 0.004 |
| Hypertriglyceridemia |  |  |  |  |  |  |
| No | Reference |  | Reference |  | Reference |  |
| Yes | 1.765 (1.442-2.16) | <0.001 | 1.355 (1.096-1.675) | 0.005 | 1.388 (1.114-1.73) | 0.003 |
| No. of Metabolic Syndrome traits |  |  |  |  |  |  |
| 0 | Reference |  | Reference |  | Reference |  |
| 1 | 1.074 (0.762-1.512) | 0.684 | 0.930 (0.653-1.323) | 0.685 | 0.868 (0.606-1.243) | 0.439 |
| 2 | 2.049 (1.475-2.846) | <0.001 | 1.584 (1.103-2.276) | 0.013 | 1.529 (1.058-2.210) | 0.024 |
| 3 | 3.039 (2.104-4.388) | <0.001 | 2.008 (1.299-3.105) | 0.002 | 1.893 (1.213-2.952) | 0.005 |
| 4 | 5.480 (3.570-8.411) | <0.001 | 2.978 (1.775-4.999) | <0.001 | 2.686 (1.577-4.574) | <0.001 |
| 5 | 4.088 (1.938-8.623) | <0.001 | 2.347 (1.040-5.298) | 0.040 | 2.260 (0.994-5.139) | 0.052 |
| P for trend | 1.523 (1.404-1.652) | <0.001 | 1.329 (1.193-1.480) | <0.001 | 1.311 (1.173-1.465) | <0.001 |
| MetS |  |  |  |  |  |  |
| No | Reference |  | Reference |  | Reference |  |
| Yes | 2.694 (2.158-3.364) | <0.001 | 1.730 (1.324-2.261) | <0.001 | 1.671 (1.270-2.199) | <0.001 |

Model 1 has no variables adjusted; Model 2 adjusted age, sex, TDI, smoking status, alcohol drinker status, education score, history of AP, history of GSD, BMI, and energy intake; Model 3 further adjusted with calcium, CRP, lymphocyte percentage, monocyte count, neutrophil count and percentage, white blood cell count, Hct, and Hb.

| Supplementary Table 3. The association between metabolic syndrome and the risk of chronic pancreatitis (Excluding 3-Year Incident Cases) | | | | | | |
| --- | --- | --- | --- | --- | --- | --- |
|  | **Model 1** | | **Model 2** | | **Model 3** | |
| Type | **HR (95% CI)** | ***P*-Value** | **HR (95% CI)** | **P-Value** | **HR (95% CI)** | ***P*-Value** |
| Hyperglycemia |  |  |  |  |  |  |
| No | Reference |  | Reference |  | Reference |  |
| Yes | 3.809 (3.119-4.652) | <0.001 | 2.614 (2.107-3.244) | <0.001 | 2.535 (2.028-3.169) | <0.001 |
| Hypertension |  |  |  |  |  |  |
| No | Reference |  | Reference |  | Reference |  |
| Yes | 1.590 (1.290-1.959) | <0.001 | 1.107 (0.885-1.384) | 0.374 | 1.090 (0.867-1.370) | 0.460 |
| Central obesity |  |  |  |  |  |  |
| No | Reference |  | Reference |  | Reference |  |
| Yes | 2.199 (1.789-2.704) | <0.001 | 1.358 (1.038-1.777) | 0.025 | 1.317 (1.000-1.733) | 0.050 |
| Dyslipidemia |  |  |  |  |  |  |
| No | Reference |  | Reference |  | Reference |  |
| Yes | 2.918 (2.385-3.569) | <0.001 | 1.592 (1.262-2.010) | <0.001 | 1.532 (1.206-1.947) | <0.001 |
| Hypertriglyceridemia |  |  |  |  |  |  |
| No | Reference |  | Reference |  | Reference |  |
| Yes | 1.650 (1.384-1.966) | <0.001 | 1.285 (1.070-1.543) | 0.007 | 1.341 (1.109-1.621) | 0.002 |
| No. of Metabolic Syndrome traits |  |  |  |  |  |  |
| 0 | Reference |  | Reference |  | Reference |  |
| 1 | 1.048 (0.766-1.432) | 0.770 | 0.879 (0.636-1.214) | 0.434 | 0.883 (0.632-1.232) | 0.463 |
| 2 | 1.988 (1.473-2.681) | <0.001 | 1.500 (1.079-2.084) | 0.016 | 1.564 (1.114-2.196) | 0.010 |
| 3 | 3.848 (2.808-5.273) | <0.001 | 2.547 (1.747-3.713) | <0.001 | 2.604 (1.766-3.842) | <0.001 |
| 4 | 6.118 (4.243-8.821) | <0.001 | 3.472 (2.226-5.417) | <0.001 | 3.452 (2.183-5.458) | <0.001 |
| 5 | 4.162 (2.240-7.736) | <0.001 | 2.304 (1.162-4.571) | 0.017 | 2.096 (1.026-4.284) | 0.042 |
| P for trend | 1.594 (1.488-1.707) | <0.001 | 1.399 (1.275-1.534) | <0.001 | 1.389 (1.263-1.527) | <0.001 |
| MetS |  |  |  |  |  |  |
| No | Reference |  | Reference |  | Reference |  |
| Yes | 3.300 (2.747-3.965) | <0.001 | 2.236 (1.783-2.803) | <0.001 | 2.184 (1.732-2.754) | <0.001 |

Model 1 has no variables adjusted; Model 2 adjusted age, sex, TDI, smoking status, alcohol drinker status, education score, history of AP, history of GSD, BMI, and energy intake; Model 3 further adjusted with calcium, CRP, lymphocyte percentage, monocyte count, neutrophil count and percentage, white blood cell count, Hct, and Hb.
